# Petechiae, Purpura, and a Pandemic: A Recipe for Scurvy

**DOI:** 10.7759/cureus.10960

**Published:** 2020-10-15

**Authors:** Charles M Seifer, Alexander Glaser, Quinto Gesiotto, Roy Waknin, Kellee L Oller

**Affiliations:** 1 Internal Medicine, University of South Florida Morsani College of Medicine, Tampa, USA

**Keywords:** scurvy, vitamin c, ascorbic acid, petechiae, purpura, bleeding, ecchymosis

## Abstract

This case report presents the case of a 28-year-old man who developed scurvy during the coronavirus disease 2019 (COVID-19) pandemic. Scurvy is a disease resulting from a nutritional deficiency of vitamin C (ascorbic acid). It is a rare condition, whose signs and symptoms can vary from patient to patient. The treatment is vitamin C supplementation, which is often followed by a swift recovery. To our knowledge, this is the first reported case of scurvy during the COVID-19 pandemic. This article highlights a rare acquired bleeding disorder, which may manifest more commonly during a pandemic due to food scarcity or stay-at-home mandates in those already at risk.

## Introduction

Scurvy is historically known as a disease that affects nutritionally depleted sailors. Today, it is uncommon in developed countries, and it is more often found in individuals of low socioeconomic status, the elderly, those who abuse alcohol, children with developmental disorders, and individuals with restrictive diets [1]. The clinical features vary greatly and may include bleeding, bruising, fatigue, musculoskeletal pain, edema, gingival symptoms, poor wound healing, weight loss, or memory impairment. With vitamin C supplementation, a full recovery is expected over a period of days to weeks [2]. Scurvy can be a difficult diagnosis to establish; therefore, it is important to maintain a high index of suspicion in at-risk individuals, especially during a period of social restrictions imposed during a pandemic.

## Case presentation

A 28-year-old man presented to the emergency department with two weeks of painful bilateral ecchymoses of his legs and flank. Additionally, he reported fatigue, shortness of breath with exertion, and lightheadedness. He had never experienced these symptoms before. He denied trauma, fever, weight loss, night sweats, cough, abdominal pain, frequency, urgency, or changes in bowel movements.

His medical history was significant for major depression, anxiety, and ADHD, which were treated with citalopram, propranolol, and dextroamphetamine/amphetamine, respectively. He denied changes to his medications in over a year. He was not taking over-the-counter medication or herbal supplements. He recently graduated from college and was living on his own for the past one and a half years. He reported a recent increase in alcohol consumption since the coronavirus disease 2019 (COVID-19) pandemic began and was drinking five to six drinks per day. He also reported intermittent marijuana use during this time. He denied any history of tobacco use or illicit drug use. His mother died from complications of type 1 diabetes. His father was alive and reported a history of monoclonal gammopathy. He denied any family history of bleeding, bruising, clotting, or hematologic malignancy. Since the pandemic and stay-at-home orders started three months ago, he reported that his diet changed from fast food and restaurant meals to home-cooked meals. He would order groceries once a month, eating mainly meat (sausage, chicken, burgers) and eggs. He denied intake of fruits, vegetables, or juice.

**Figure 1:**
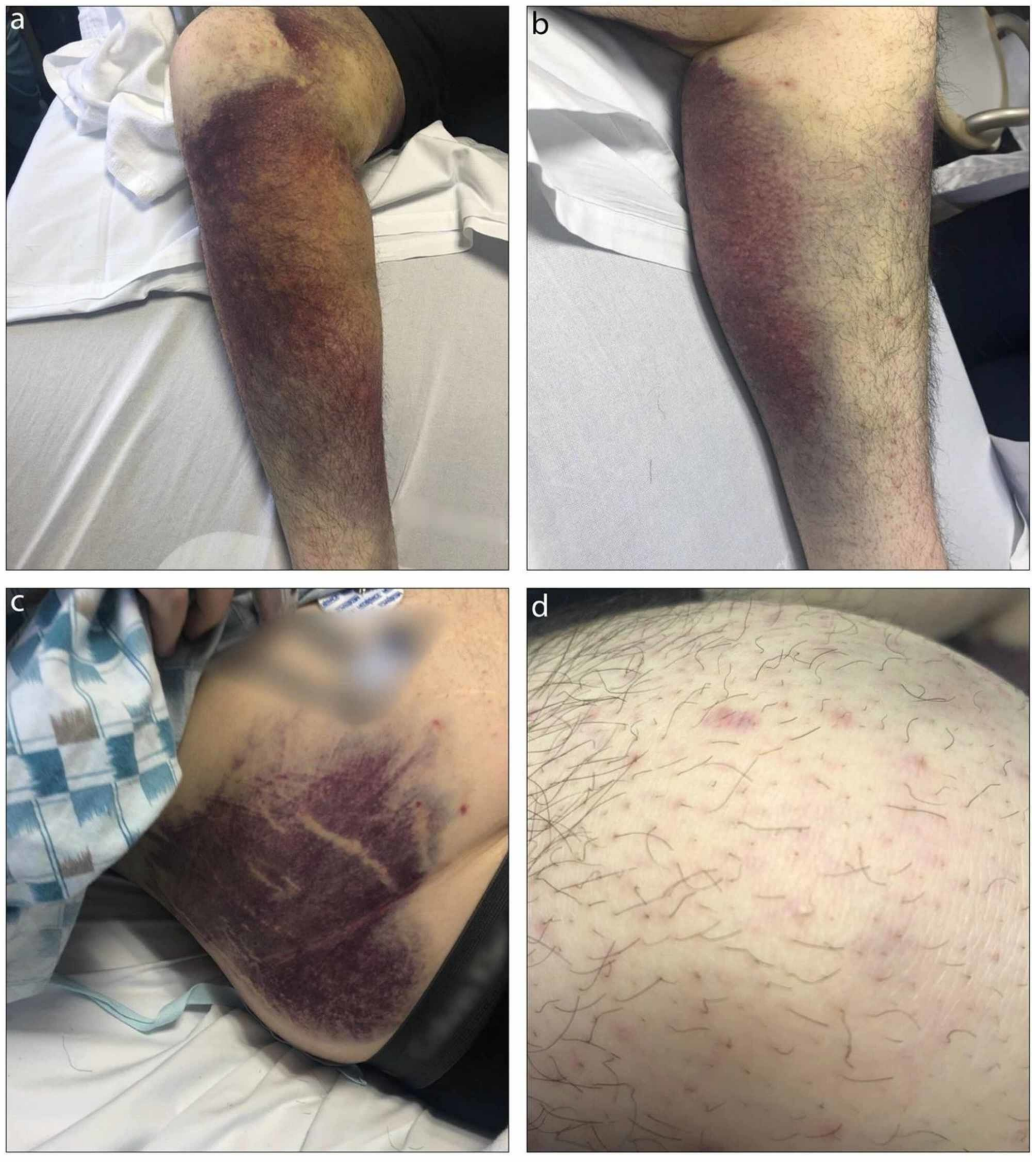
A 28-year-old man who presented with painful ecchymoses of his legs and flank bilaterally. (a,b) Bilateral legs with dense ecchymoses. (c) Right flank with significant ecchymosis. (d) Closer examination revealed petechial distribution around the hair follicles and corkscrew hairs.

On physical examination, his temperature was 99.1°F, pulse was 97 beats per minute, respiratory rate was 19 breaths per minute, blood pressure was 112/80 mmHg, and oxygen saturation was 97%. His BMI was 32.83 kg/m^2^. He was slightly anxious and lying flat in bed. The legs were covered in bilateral ecchymoses, non-palpable purpura, and petechiae (Figures 1a, 1b). There were also large ecchymoses on both flanks (Figure 1c). He had conjunctival pallor and slight scleral icterus, as well as dry mucous membranes and poor dentition. There was no evidence of gingival bleeding. The liver and spleen were unable to be palpated due to body habitus. Closer examination of the skin revealed perifollicular petechiae and corkscrew hairs (Figure 1d).

His laboratory values revealed hyponatremia, hypokalemia, and hypochloremia. He was anemic with hemoglobin of 8.4 g/dL and mean corpuscular volume (MCV) of 91.7 fL. Folate levels were reduced. White blood cell count and platelets were slightly elevated at 12.1 k/mm^3^ and 476 k/mm^3^, respectively, which returned to normal levels following hydration. Lactate dihydrogen (LDH) was elevated at 614 U/L, and total bilirubin was elevated at 3.2 mg/dL. Fibrinogen was elevated at 563 mg/dL. D-dimer was elevated at 13.7 mg/L. Prothrombin time (PT) was slightly prolonged at 14.6 seconds and partial thromboplastin time (PTT) was normal at 26.9 seconds. Haptoglobin was normal at 155 mg/dL. Von Willebrand factor (VWF), factor VIII activity, and platelet function assay were all within normal limits. Peripheral smear demonstrated bands, nucleated red blood cells, and megakaryocytes, with no evidence of blasts or schistocytes. Reticulocyte count was increased to 11.6.

His hemoglobin fell to 6.6 g/dL, and he was given one unit of packed red blood cells. His hemoglobin levels subsequently increased, and his fatigue, lightheadedness, and shortness of breath improved. A vitamin C level was collected, and he was started on high-dose vitamin C therapy for suspected deficiency. Subsequently, his vitamin C level returned undetectable (<0.1 mg/dL).

**Figure 2:**
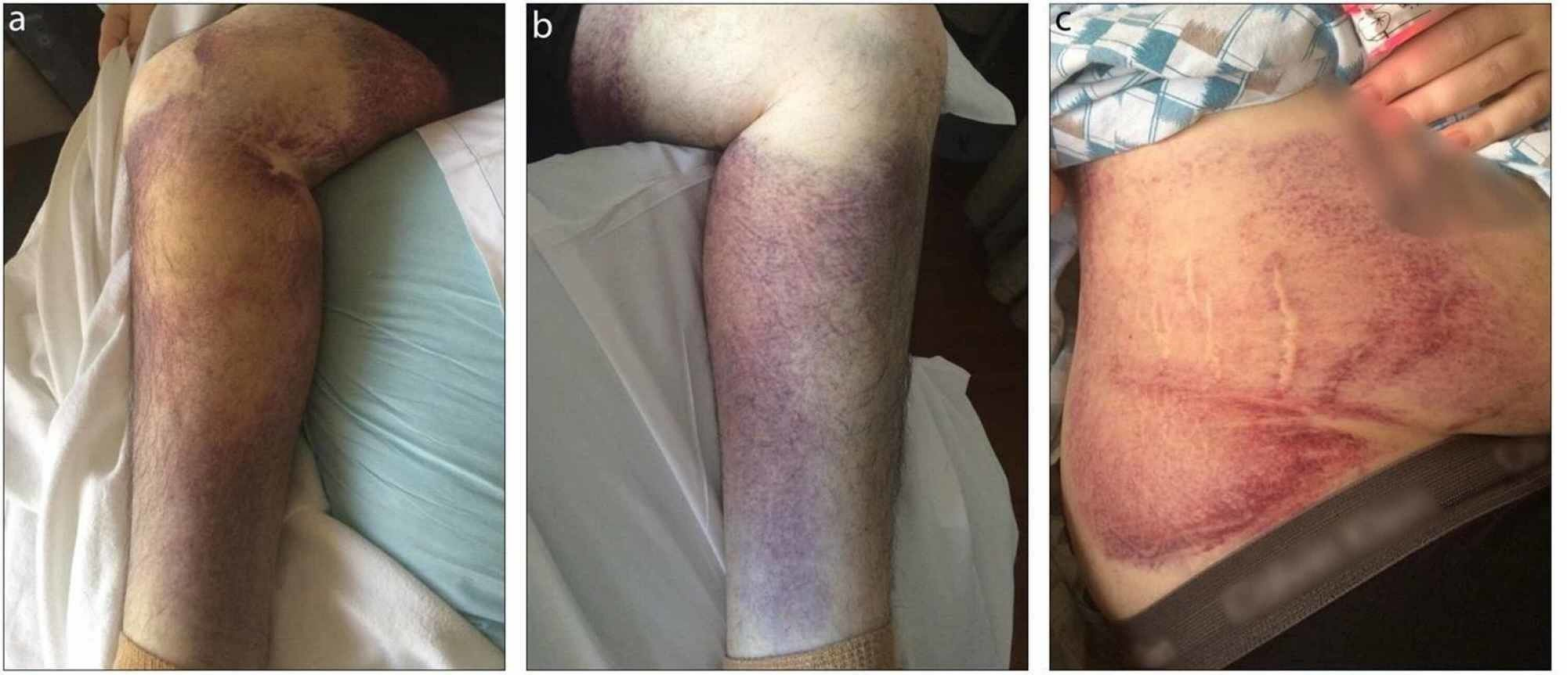
Ecchymoses three days post-treatment with 1 g IV vitamin C. (a,b) Bilateral legs with ecchymoses. (c) Right flank ecchymosis.

Our patient was treated with 1 g IV vitamin C for three days. His bleeding, leg pain, and bruising all improved (Figures 2a-2c), and he was discharged on oral supplementation of vitamin C 250 mg daily in addition to nutritional education.

## Discussion

Given the low prevalence and heterogeneous clinical presentation, the diagnosis of vitamin C deficiency (scurvy) can be elusive. Bleeding and bruising are the most common clinical features [2]. The differential for bleeding disorders is broad and includes etiologies of primary hemostasis (thrombocytopenia and platelet dysfunction) as well as secondary hemostasis (coagulation factor deficiency or inhibitors, for example). The differential in patients with normal platelet counts and coagulation parameters is much more focused and includes disorders such as von Willebrand disease, connective tissue disorders (such as Ehlers-Danlos syndrome), mild clotting factor deficiencies, platelet function disorders, and scurvy [2-4].

Ascorbic acid, an essential nutrient, is unable to be synthesized endogenously by the human body and must be obtained from dietary sources. Recommended daily consumption for an adult male is 75-90 mg [5]. Ascorbic acid has multiple functions within the body, particularly as a cofactor in the synthesis of collagen [6]. Defective collagen synthesis manifests as impaired wound healing and poor dentition. Symptoms often present when plasma concentrations fall below 0.2 mg/dL (normal = 0.2-2 mg/dL) [1,7]. It can take between one and three months of inadequate nutrition for symptoms to appear [2]. The first documented case of COVID-19 in Florida was on March 1, 2020 [8]. Widespread closure of public schools, restaurants, and beaches occurred within the next few weeks, and an official stay-at-home order was issued in April 1, 2020. Our patient was admitted three months later.

There are no official criteria for diagnosing scurvy. Evidence of vitamin C (ascorbic acid) deficiency and clinical manifestations are sufficient. Historically, scurvy was a devastating disease that claimed the lives of many explorers and sailors. Today, scurvy is uncommon in the United States, although certain populations continue to be at risk. There are many case reports of scurvy in the elderly patients over the age of 65 years [1,3,7,9]. These patients were often malnourished, financially limited, and/or used alcohol excessively. Recent studies have also highlighted increased risk of scurvy in children with autism [10-11]. Children with autism spectrum disorder may have high food selectivity, making them prone to nutritional deficiencies. Finally, certain diets are associated with cases of scurvy. The ketogenic diet (low carbohydrate, high fat) has been linked with vitamin C deficiency in patients with epilepsy [12-13]. Ultimately, one should have a high index of suspicion for scurvy in patients with bleeding or bruising abnormalities with risk factors including unsteady food supply, food aversion, restrictive or ketogenic diets, or chronic alcohol use. To our knowledge, this is the first reported case of scurvy related to the COVID-19 pandemic altering a typical diet. On a population-wide level, extenuating circumstances such as increased social isolation, job loss, travel restrictions, and restaurant closures may increase the risk for malnutrition in otherwise healthy people.

Unsurprisingly, vitamin C supplementation is the best treatment for scurvy. Various dosage recommendations have proven effective. With treatment, the prognosis is good. Spontaneous bleeding usually stops within one day. Oral lesions can heal in two to three days. Ecchymoses usually heal within 12 days [2].

## Conclusions

Scurvy develops in individuals who are vitamin C deficient. While uncommon, it should be included in the differential of those who present with bleeding or bruising. This is especially true in groups at increased risk of nutritional deficiency, especially in a pandemic where resources, travel, and communication may be limited. Fortunately, the prognosis is excellent with vitamin C supplementation.
